# Effect of transbronchial or intravenous administration of indocyanine green on resection margins during near-infrared-guided segmentectomy: a review

**DOI:** 10.3389/fsurg.2024.1430100

**Published:** 2024-07-01

**Authors:** László Libor, Balázs Pécsy, Evelin Szűcs, Judit Lantos, Annamária Bakos, György Lázár, József Furák

**Affiliations:** ^1^ Department of Surgery, Faculty of Medicine, University of Szeged, Szeged, Hungary; ^2^ Department of Neurology, Bács-Kiskun County Hospital, Kecskemet, Hungary; ^3^ Department of Nuclear Medicine, Faculty of Medicine, University of Szeged, Szeged, Hungary

**Keywords:** lung surgery, near-infrared fluorescence-guided surgery, indocyanine green, segmentectomy, VATS, resection margin, intersegmental plane

## Abstract

For early-stage non-small cell lung cancer, surgical resection remains the best treatment option. Currently, sublobar resection, including segmentectomy, is recommended in these cases, as it provides a better quality of life with the same oncological outcomes; however, is requires adequate resection margins. Accurate preoperative planning and proper identification of the intersegmental planes during thoracic surgery are crucial for ensuring precise surgical management and adequate resection margins. Three dimensional computed tomography reconstruction and near-infrared-guided intersegmental plane identification can greatly facilitate the surgical procedures. Three-dimensional computed tomography reconstruction can simulate both the resection and resection margins. Indocyanine green is one of the most frequently used and affordable fluorophores. There are two ways to identify the intersegmental planes using indocyanine green: intravenous and transbronchial administration. Intravenous application is simple; however, its effectiveness may be affected by underlying lung disease, and it requires the isolation of segmental structures before administration. Transbronchial use requires appropriate bronchoscopic skills and preoperative planning; however, it also allows for delineation deep in the parenchyma and can be used for complex segmentectomies. Both methods can be used to ensure adequate resection margins and, therefore, achieve the correct oncological radicality of the surgical procedure. Here, we summarise these applications and provide an overview of their different possibilities.

## 1 Introduction

Surgical resection remains one of the best therapeutic options for the long-term survival of patients with early stage non-small cell lung cancer (NSCLC) (1). Multicentre trials have shown that there is no significant difference in 5-year survival between lobectomy and segmentectomy or sublobar resection in early-stage (Stage IA) NSCLC, where the tumour size is ≤2 cm. In the JCOG0802/WJOG4607l trial, the 5-year relapse-free survival rates were 87.9% and 88.0%, respectively, while the 5-year overall survival rates were 91.1% and 94.3%, respectively, for lobectomy and segmentectomy. In the CALGB clinical trial, the 5-year disease-free survival rates were 64.1% and 63.6%, and the 5-year overall survival rates were 78.9% and 80.3%, respectively (lobectomy vs. sublobar resection). Notably, the proportion of anatomical segmentectomies among sublobar resections in this study was 37.9% compared to 59.1% for wedge resections. However, the incidence of locoregional recurrence was significantly higher in patients who underwent segmentectomy (11% vs. 5%, segmentectomy vs. lobectomy *P* = 0.0018) in the JCOG0802/WJOG4607l trial. Similarly, in the CALGB trial, locoregional recurrence was observed in 10.0% of the lobectomies and 13.4% of the sublobar resections (2, 3). Importantly, tumours located in the periphery of the lungs (an indication for segmentectomy) may have a higher locoregional recurrence rate (4).

Due to the development of a variety of national screening programs and sensitive diagnostics, an increasing number of early stage lung cancers are being detected (5). With the increase in minimally invasive surgical procedures, video-assisted thoracic surgery (VATS) (6, 7) and robot-assisted thoracic surgery (8) have emerged and are now the preferred procedures for early stage lung cancer surgery, providing better perioperative outcomes with reduced postoperative pain, improved quality of life, and equivalent oncological outcomes compared to open surgery.

Despite the use of more complex surgical techniques, such as the identification of intersegmental planes (ISP), the complication rate was comparable between VATS segmentectomies and lobectomies, and the length of stay and drainage time were reduced for segmentectomies (9). Patients with early stage NSCLC (≤2 cm) localised in the outer third of the parenchyma are candidates for segmentectomy. Accordingly, the European Society of Thoracic Surgeons has formulated recommendations for segmentectomy for early NSCLC (10).

Inadequate resection margins and lymph node removal are the main causes of locoregional recurrence. Therefore, ensuring and defining adequate margins and lymph node status are crucial for reducing local recurrence rates in segmentectomies (2, 3). To ensure adequate resection margins, it is important to identify the ISP.

The standard approach for segmentectomy is to perform vascular and bronchial transections, and then divide the ISPs. There are different preoperative and intraoperative methods for tumour localisation and identification of the ISPs. Chest computed tomography (CT) and its three-dimensional (3D) reconstruction are important in preoperative planning. A virtual bronchogram and accurate identification of vascular structures, segments, or planes can make the procedure safer and allow for complete resection (11). Near-infrared (NIR)-guided surgery is a relatively new technique that makes it easier to identify the ISPs. Indocyanine green (ICG) can be used to visualise segmental boundaries, eliminating the need for traditional inflation or deflation methods, and can be administered intravenously or via endobronchial injections (12, 13).

Using fluorophores, the localisation of lesions and their intraoperative visualisation can be used to determine the proper resection margins. Pafolacianine is a novel fluorophore used for intraoperative molecular imaging (IMI) (14). In this narrative review, we discuss the advantages and disadvantages of ICG administration methods by comparing the characteristics of different applications, and the role of preoperative 3D CT reconstruction.

## 2 Methods

This narrative review aims to discuss the relevant literature regarding the different applications of ICG, and the impact of NIR-guided surgery on the resection margins during thoracoscopic segmentectomy in early-stage NSCLC. We searched the literature on PubMed. The search was narrowed to articles between 2017 and 2023. The keywords used in the search were “thoracoscopic segmentectomy”, “near-infrared fluorescence”, “indocyanine green”, “resection margin”, “transbronchial”, “endobronchial” and “intravenous”. Eventually, after reviewing the abstracts, 8 articles were selected for analysis. The following questions were investigated in the articles we reviewed. What are the advantages and disadvantages of different applications of ICG in NIR-guided lung surgery? How does NIR fluorescence imaging help to achieve adequate resection margins in early-stage NSCLCs? How does preoperative 3D CT reconstruction facilitate surgical planning? After reviewing the articles, we summarized the information that we found relevant in the form of text and tables.

## 3 Preoperative planning: the role of 3D CT reconstruction

To perform lung segmentectomy, the surgeon must be familiar with the radiologic segmental anatomy. Preoperative review of chest CT scans helps to localise the nodule and determine the anatomy of the segment, mapping the bronchi and blood vessels. High-resolution CT scans provide an accurate picture of the segmental anatomy and 3D reconstructions help to accurately define the tumour and segmental structures and visualise bronchovascular variations. It is essential to identify the arteries and bronchi of the target segment, as well as the intrasegmental veins, and to preserve the intersegmental veins as a prerequisite for identifying the intersegmental planes. This is greatly facilitated by appropriate 3D reconstruction (15). Additionally, segmentectomies can be simulated using virtually defined intersegmental planes and resection margins. Together, these methods facilitate surgical planning and help to prevent locoregional recurrence by aiding in defining the resection margins (2, 16).

Most authors use thin-slice (1 mm) chest CT with intravenous contrast. For 3D reconstruction, the two software packages “Synapse Vincent” (Fujifilm, Tokyo, Japan) and “Ziostation2” (Ziosoft, Inc., Tokyo, Japan) are often used. These scans are used to automatically reconstruct the vasculature, bronchial tree, and parenchyma. In addition, tumour localisation, adjacent anatomical structures, virtual intersegmental planes, resection margins, and segment volumes can be determined in collaboration with the surgeon. One of the most important applications of CT is planning the resection margin. The shortest distance between the tumour and planned resection line was determined using reconstruction software. The nearest resection margin was defined as being at least 2 cm from the tumour or greater than its diameter. Resection distances were defined according to the recommendations of Sawabata et al. (17). If this was less, the planned resection was extended to the adjacent segments. Thus, complex or multiple segmentectomies can be planned. The concordance rate between the simulated and specimen resection margins and between segmental structures was 90% or above (18–20).

Chang et al. performed intraoperative cone-beam CT in addition to preoperative 3D CT planning. This rare procedure requires a hybrid operating room. During NIR guidance (intravenously administered ICG), the defined intersegmental planes were marked with metal clips and verified during the CT scan and 3D reconstruction. The resection margins were then calculated. Intraoperative CT scan was performed under bilateral ventilation. The planned resection margins were determined by the distance between the tumour, the intersegmental planes marked with metal clips, and the planned stapler line. If the calculated resection margin was less than 2 cm, the resection was extended to adjacent segments. The median time of the intraoperative CT scan was 10.5 min despite the complexity of the procedure. The concordance rate between intraoperatively reconstructed 3D CT scans and preoperatively simulated scans was 97.1% (21). The correlation and/or difference between the calculated preoperative and intraoperative resection margins and the resection margins measured on the specimens are shown in Table 1.

**Table 1:** Characteristics of the preoperative computed tomography reconstruction and simulation.

Study	Sekine et al. (18)	Wada et al. (22)	Sekine et al. (20)	Matsuura et al. (19)	Sun et al. (23)	Chang et al. (21)	Guigard et al. (24)	Sun et al. (15)
Year	2019	2020	2021	2022	2022	2023	2017	2019
No. of patients	65	15	28	20	35	34	22	19
3D reconstruction	Yes	Yes	Yes	Yes	Yes	Yes	Yes	Yes
Preoperative margin simulation (mm)	21.5 ± 11.2	N/A	11.4 ± 5.4	30.5 ± 10.1	N/A	N/A	N/A	N/A
Intraoperative margin simulation (mm)	N/A	N/A	N/A	N/A	N/A	15.4 ± 7.2	N/A	N/A
Pathological margin (mm)	23.5 ± 8.3	100% complete resection	12.2 ± 4.1	31.0 ± 11.0	20.7 ± 5.9	23.1 ± 8.3	100% complete resection	100% complete resection
Significance of the difference	*P* = 0.19	N/A	*P* = 0.285	*P* = 0.801	N/A	N/A	N/A	N/A
Concordance rate	93.10%	N/A	N/A	90%	N/A	97.1% (preoperative-postoperative)	N/A	N/A
ICG administration	Trans-bronchial	Trans-bronchial	Trans-bronchial	I.V.	Peri-tumoral/I.V.	I.V.	I.V.	I.V.

N/A, not applicable; I.V, intravenous.

Wada et al. performed preoperative volume measurements during 3D CT reconstruction. The entire lung was scanned during a single breath hold at the end of inspiration. The volume of the ICG injected endobronchially was calculated after determining the volume of the target segment. The ICG volumes were then used during NIR-guided surgery; no adverse events were recorded (section 3.4). The volume of the ICG administered endobronchially was at least 0.04 of the target segment volume. Virtual bronchoscopy performed during reconstruction also supported the selection of bronchi for ICG administration in the segment of interest (22).

## 4 Near-infrared guided sublobar resection

### 4.1 NIR and ICG

Invisible NIR fluorescence imaging has been used over the last two decades to assist with intraoperative findings. Since this technique does not require ionising radiation, it is safe (25). Further, because NIR light is invisible to the human eye, it does not affect the surgeon's vision. NIR fluorescence imaging systems are based on the detection of infrared light emitted by fluorescent dyes. A special camera is used for this purpose after the tissues are excited by a specific infrared light. All of these systems can be integrated into cameras used in open and mostly laparoscopic or robotic surgeries. NIR imaging systems offer several advantages. While visible light can penetrate only a few micrometres into tissues, NIR light (700–900 nm) can penetrate centimetres through different tissues (26).

Several NIR systems can detect infrared light. These systems are affordable and simple to use and can be used as alternatives to conventional, complicated, and expensive intraoperative imaging techniques. Fluorophores are required in addition to NIR light detectors. The most commonly used fluorophore is ICG, a water-soluble, amphiphilic tricarbocyanine fluorophore. It has a molecular weight of 775 D and it fluoresces and absorbs the NIR spectrum. ICG binds rapidly to plasma proteins and lipoproteins. It is secreted through the liver into bile in an unconjugated form, is not metabolized, and has a half-life of 120–240 s (27).

ICG is safe for systemic use up to a dose of 5 mg/kg. The excitation and emission wavelengths are approximately 805 and 830 nm, respectively. With an emission spectrum at 830 nm, the tissue penetration of ICG is up to 15 mm. Owing to its amphiphilic and protein-binding characteristics, it can migrate through lymphatic vessels. ICG is relatively inexpensive, nontoxic, and approved by the Food and Drug Administration. It is affordable, making it an optimal fluorophore for NIR-guided surgery. ICG has long been used in cardiac function tests, functional liver examinations, and ophthalmic angiography (28, 29).

However, ICG has some disadvantages, such as moderate photostability, a relatively narrow fluorescence quantum yield, high propensity for binding to plasma proteins, and aggregation in aqueous solutions (12). Some cases of anaphylaxis have also been reported during its use and it is not recommended for patients with iodine allergy or thyreotoxicosis (30, 31).

Pafolacianine, also known as OTL38, is a newly developed ICG-like folate analogue. Binding to the folate receptor alpha can be used to mark folate receptor-positive tumours. It is eliminated relatively quickly from receptor-negative tissues, with a half-life of <30 min (32, 33). NIR-guided lung surgery is suitable for localising lesions, considering that 85% of lung tumours express folate receptors (34, 35) that are unaffected by chemotherapy (36). Besides rapid plasma clearance, it also has several additional benefits, such as a long residence time in the tumour, which allows it to be administered on the day of surgery in cases of active tumour targeting. Furthermore, pafolacianine has a better penetration depth and signal-to-background ratio and was approved by the Food and Drug Administration in late 2022 (14, 36).

### 4.2 Determination of intersegmental planes

Delineation and division of the intersegmental planes are critical in segmentectomy. These steps ensure appropriate oncological radicality by securing the resection margins but can also lead to a decision to extend the resection if necessary. One method often used to determine the ISPs is the inflation-deflation technique or it's modification. This technique involves re-ventilating the lungs after clamping the target bronchus and this is performed as described below: After identification and ligation of the targeted segmental bronchus, artery and intrasegmental vein, the collapsed lung is fully re-expanded with controlled airway pressure below 20 cmH2O, opening the bronchus on the operated side to the atmosphere while continuing the ventilation of the contralateral lung. Five to twelve minutes later, a demarcation forms between the inflated target segment and adjacent deflated segments, representing the ISP (37). However, it is often inaccurate due to collateral ventilation and is difficult to use in patients with underlying lung diseases such as emphysema. In addition, during VATS, the surgeon's vision is disturbed by the inflated lungs. Selective ventilation of the target bronchus can also be performed, although it is a relatively complicated procedure (38).

In 2009, a new method was introduced to identify ISPs. In this method, an NIR thoracoscope is used after the intravenous administration of ICG (12, 39). Using the surface blood flow of the lung, the segments can be visualised after the bronchi, and the vessels of the targeted segment are clamped. This method is currently widely accepted (40).

Endobronchial administration is another option for the use of ICG. This is a relatively complex bronchoscopic procedure. These methods allow for the delineation of target segments based on the distribution of ICG in the lungs. These application methods have advantages and disadvantages, as explained below (13).

Many factors can influence the delineation of ISPs. Therefore, a delineation score is sometimes used to describe the degree of ISP identification. In general, the detectability of ISP is at least 90% with the use of ICG (18, 19).

### 4.3 Intravenous administration of ICG

Since 2009, when Misaki et al. reported the benefits of intravenously administered ICG during thoracoscopic surgery, this method has been widely used (39). In general, ICG is administered intravenously after the identification and division of the bronchi and vessels of the targeted segment or segments previously identified during 3D CT reconstruction and simulation. Staining usually takes 200–400 s after injection. Using an NIR thoracoscope, the visceral pleura shows fluorescence, except in the affected segment. This method is referred to as negative staining.

After 3D CT reconstruction and planning, Matsuura et al. used ICG at a dose of 0.3 mg/kg/10 ml administered at a rate of 300 ml/h. The demarcation was evaluated by two authors, and each intersegmental plane was scored as follows: clear visualisation without interruption (3 points, excellent), partially unclear visualisation but can be identified (2 points, fair), and difficult to identify (1 point, poor). The demarcation was marked with electrocautery and divided using staplers. In total, 92.2% of the 64 ISPs examined were visualised. All the tumours were completely removed. This method is now considered the standard for intravenously administered ICG (19), including the use of staplers for division of the parenchyma (41).

In several studies, 100% successful ISP delineation has been achieved with intravenous administration of ICG (15, 20, 23, 24), although the lack of emphysema may facilitate this success rate. In the study by Sun et al., the ISPs determined by ICG was fully consistent with those determined by the modified inflation-deflation method. At the same time, ICG application saves the waiting time required for the modified inflation-deflation method, and also avoids complications associated with the procedure, such as pressure trauma (15). Intravenous ICG may affect the extent of the resection. Thus the preoperatively planned anatomical resection can be modified during NIR angiography. Dividing the intersegmental planes determined by the vasculature would prevent retention of devascularized parenchyma. Adequate management of vascular variations detected in the background of inappropriate delineation, can avoid devitalisation of adjacent segments, or can ensure adequate resection margins by extending the resection if necessary. During surgery, we can freely choose pulmonary artery branches, which can be clamped to change the extent of the resection. Accordingly, we have the possibility to modify the resection margin during surgery if it is considered insufficient. In this way, oncological safety can be increased. The repeatability and quickness of intravenous administration is a real advantage in this case (19, 24). In the study by Guigard et al., the use of intravenously administered ICG changed the progress of segmentectomy in three of the 22 cases (24).

However, assessment of the ISPs delineated after ICG administration is not always straightforward and can be influenced by a number of factors. In approximately 10% or less of the cases, the ISPs cannot be visualised (39, 42). In patients with severe chronic obstructive pulmonary disease, emphysematous lungs, or anthracosis, the possibility of identifying the intersegmental line could be reduced (43). This implies that insufficient resection margins may be achieved during surgery under certain circumstances. Therefore, it is necessary for surgeons to confirm that the ISP to be drawn matches the actual anatomical or simulated line during preoperative planning. Intraoperative CT scanning and 3D reconstruction may be potential solutions to this problem. Chang et al. used this method. After intravenous ICG administration (5 mg/patient) followed by bronchovascular clamping and delineation, the intersegmental planes were marked with metal clips, and intraoperative cone beam CT 3D reconstruction was performed to verify the relationship between the ISP and the tumour. The intersegmental planes were well defined by ICG in 91.2% of the cases. The concordance between CT reconstructions was similarly high. This intervention provided confirmation to the surgeon or provoked extension of the surgery when needed. Intraoperative CT was performed under bilateral ventilation, and the median time required for intraoperative CT imaging was 10.5 min (21).

Marking methods are another option for intraoperatively identifying tumours and ensuring proper radical resection. CT-guided hook-wire markings (44), coil markings (45), transbronchial fiducial markers, and dye injections may all be suitable for this purpose (46).

Sun et al. used CT-guided percutaneous ICG injection paratumorally immediately before surgery to localise nodules intraoperatively. A 0.1 ml dose (2.5 mg/ml concentration) was injected into the medial border of the tumour. The average time to nodulus localisation was 15.48 ± 5.13 min. Otherwise, intravenous ICG was administered and negative staining was performed intraoperatively. As a result, dual visualisation was achieved, which allowed for real-time monitoring of the tumour-ISP relationship. This ensured an adequate resection margin. However, this method is not suitable for marking deep nodules owing to its NIR penetration abilities. It is an invasive procedure that must be closely synchronised with the timing of surgery (23). Further characteristics of the applications are shown in Table 2.

**Table 2:** Characteristics of ICG administration and postoperative outcomes.

Study	Sekine et al. (18)	Wada et al. (22)	Sekine et al. (20)	Matsuura et al. (19)	Sun et al. (23)	Chang et al. (21)	Guigard et al. (24)	Sun et al. (15)
Year	2019	2020	2021	2022	2022	2023	2017	2019
No. of patients	65	15	28	20	35	34	22	19
ICG administration	Trans-bronchial	Trans-bronchial	Trans-bronchial	I.V.	Peritumoral/I.V.	I.V.	I.V.	I.V.
ICG dose	2.5 mg/10 ml	1.25 mg/10 ml	2.5 mg/10 ml	0.3 mg/kg	5 mg	0.3 mg/kg	12.5mg	5 mg
ISP delineation (%)	89.2%	87%	100%	92.2%	100%	91.2%	100%	100%
Operation time (min)	212 ± 53.0	193 ± 41	69.9 ± 19.7	129 ± 34	99.43 ± 17.4	193 ± 60.8	137.2 ± 31.4	101 ± 25.8
No. of complex or extended segmentecto-my	38 (58.5%)	6 (40%)	N/A	6 (30%)	10 (28.6%)	N/A	12 (54.5%)	8 (42.1%)
Blood loss (ml)	108.9 ± 86.0	110 ± 101	24.3 ± 43.6	15 ± 21		35 ± 72.3		43.4 ± 37
Chest drainage (days)	N/A	3.1 ± 1.0	N/A	1.1 ± 0.3	3.46 ± 1.15	2.3 ± 1.5	N/A	3.5 ± 2.1
Complications	4 (air leak, pneumonia, atrial fibrillation)	2 (air leak, cerebral infarction)	2 (air leak)	1 (PTX)	1 (air leak)	No	4 (air leak, gastroparesis, ileus)	3 (air leak, pulmonary infection)
Hospital stay (days)	8.4 ± 5.7	N/A	5.3 ± 2.3	2.3 ± 0.4	4.57 ± 1.33	N/A	5.4 ± 4.5	4.79 ± 2.02
Pathological margin (mm)	23.5 ± 8.3	100% complete resection	12.2 ± 4.1	31.0 ± 11.0	20.7 ± 5.9	23.1 ± 8.3	100% complete resection	100% complete resection

N/A, not applicable; I.V, intravenous; ICG, indocyanine green; ISP, intersegmental plane.

### 4.4 Transbronchial administration of ICG

ICG can also be used transbronchially to identify intersegmental planes. This technique is less common than intravenous application. Transbronchial injection of ICG can be performed intraoperatively following ligation of the segmental vessels and bronchi. This method was described by Oh et al., who used a generally open approach during video-assisted surgery. This manoeuvre allowed for the visualisation of the ISP; however, in contrast to intravenous application, it was not only delineated on the surface, but also deeply in the parenchyma. This is one advantage of the proposed method. However, this technique has not become popular because it requires the separation of hilar structures prior to administration and can be difficult to perform during thoracoscopic surgery (47).

Another common transbronchial method is bronchoscopic administration. This procedure is typically performed using an ultrathin bronchoscope. Thus, it is possible to administer the ICG to both the segmental and subsegmental bronchi. In all cases, precise 3D CT reconstruction and virtual bronchoscopy were used to simulate the planned resection. Reconstruction was performed up to 4th to 6th levels of the bronchi. This allowed for the accurate planning of bronchoscopic administration. In some cases, the volume of the lung segment was calculated to determine the amount of the ICG solution administered (22).

After the induction of general anaesthesia, bronchoscopy was performed through a single-lumen endotracheal tube or laryngeal mask. An ultrathin bronchoscope was used to identify the target bronchus or bronchi, followed by a bronchial catheter with a balloon to deliver the ICG solution and 50–400 ml of air into the segment. In this step, it is crucial to prevent ICG leakage as it can lead to false results if transferred to other bronchi. Positive end-expiratory pressure ventilation was initiated. As ICG binds rapidly to plasma proteins, it is dissolved in saline and autologous blood, and this solution is injected into the bronchi. On average, ICG administration took 5–22 min, and it took 15–30 min until the start of surgery (20, 22).

During VATS segmentectomy, an NIR thoracoscope was used to visualise the resection borders. The visceral pleura was marked with diathermy. The intersegmental planes were separated using electrocautery and/or a stapler. After the resection, an NIR thoracoscope was used to identify the residual tissues that should be resected. This is one of the advantages of this method because fluorescence can be observed deep in the parenchyma. The types of resection include subsegmental resection, simple segmentectomy, complex segmentectomy, and extended segmentectomy (18, 20, 22).

The concentration of the injected ICG solution was between 1.25 mg/10 ml and 2.5 mg/10 ml. The injected volume depended on the calculated segmental volume or 10 ml per subsegmental bronchi. The corresponding demarcation rate shown by ICG was 87%–100%. Sekine et al. used a four-degree scoring system to describe the degree of delineation. Complete resection was achieved in all cases, and no ICG-induced adverse reactions were reported (18, 20, 22).

Sekine et al. analysed the residual segmental structure 6 months later during a control CT scan and reconstruction and found 93.1% concordance with the preoperative simulation. Furthermore, a comparison with a traditional segmentectomy control group was performed to assess the efficacy of ICG. Complicated sublobar resection was performed more often in the ICG group (*P* < 0.0001) and the operative time was longer. However, after propensity score matching, the results were similar (18). Additional characteristics of the interventions are presented in Table 2.

## 5 Pafolacianine: the ELUCIDATE trial

Aside from ICG, other options are emerging for intraoperative imaging. In ELUCIDATE, a multicentre phase 3 trial, intravenous pafolacanine was used for active tumour targeting using IMI. Thus, it is possible to localise non-palpable tumours and detect additional lesions, metastases, and positive lymph nodes. As it is a preoperative intravenous injection, there is no need for the isolation of segmental structures prior to application or for complex additional procedures such as bronchoscopy. In the trial, after visualising the tumour using NIR imaging, it was possible to measure the resection margins *in situ* or on the back table. The IMI group comprised 100 patients. Overall, IMI with pafolacianine changed the scope of the surgical procedure in 29% of cases. A total of 65 clinically significant events occurred in 53 patients during the trial. Of these, 38 events affected the resection margins. This represents, that the most common clinically significant event was the identification of close margins. The sensitivity for detecting cancerous tissues was 76.9%. No serious adverse drug-related effects were reported (48).

## 6 Discussion

NIR-guided surgery is a relatively new technique that has been rapidly adopted in the field of thoracic surgery. The most commonly used fluorophore is ICG (25, 28, 29). Achieving adequate resection margins is one of the most crucial factors in preventing local recurrence (2, 3); this can be facilitated by the combination of three-dimensional CT reconstruction and NIR-guided surgery. Reliable visualisation of the ISP is crucial for accurate single or complex segmentectomy. In this regard, there are two ways to use ICG: intravenous, and trans-, or endobronchially. The advantages of intravenous ICG include easy and quick application and repeatability. Thus, NIR angiography can be used to modify the extent of the resection during surgery (24). Therefore, it is widely accepted. The disadvantages include a relatively short duration of fluorescence and the need to separate the vessels and bronchi of the target segment prior to administration (19, 21, 24). Furthermore, underlying lung diseases (e.g., emphysema and chronic obstructive pulmonary disease) affect the applicability of intravenous ICG (43).

In contrast, after transbronchial administration of ICG, fluorescence can be observed for a long time and detected deep in the parenchyma. Complex sublobar resections are more feasible when using virtual bronchial maps. Delineation of the ISP can be observed at the beginning of surgery, and there is no need for dissection of segmental structures before surgery (20). Disadvantages include the complexity of bronchoscopic application, which requires advanced skills and must be synchronised with the timing of the surgery. Furthermore, the ICG solution that leaks during injection may provide a false result if it is transferred into the adjacent bronchi (22).

Our findings revealed that ISP identification has similar efficacy for both methods, and overall, there were no significant differences in the duration of surgery. Complete resection was achieved in all studies, independent of the ICG application method. Limitations of the studies included a limited number of cases and short follow-up period (19, 21). Sekine et al. noted that one patient died of multiple distant metastases one year after surgery. No local recurrences were reported (20). Transbronchial administration may be advantageous in the management of complex segmentectomies. However, intravenous administration is simple and widespread.

Overall, both methods (intravenosus and transbronchial administration) can help to ensure adequate resection margins for segmentectomy in patients with early stage NSCLC. However, owing to the emergence of new fluorophores, such as pafolacianine, for active tumour targeting and IMI, the localisation of lesions can be more easily performed to provide sufficient resection margins independent of ISP determination (48). However, this requires further investigation.
